# The Mechanosensory Role of Primary Cilia in Vascular Hypertension

**DOI:** 10.1155/2011/376281

**Published:** 2011-06-16

**Authors:** Surya M. Nauli, Xingjian Jin, Beerend P. Hierck

**Affiliations:** ^1^Colleges of Medicine, Pharmacy and Pharmaceutical Sciences, The University of Toledo, Health Science Campus, HEB 274, 3000 Arlington Avenue MS 1015, Toledo, OH 43614, USA; ^2^Department of Anatomy and Embryology, Leiden University Medical Center, P.O. Box 9600, 2300 RC Leiden, The Netherlands

## Abstract

Local regulation of vascular tone plays an important role in cardiovascular control of blood pressure. Aside from chemical or hormonal regulations, this local homeostasis is highly regulated by fluid-shear stress. It was previously unclear how vascular endothelial cells were able to sense fluid-shear stress. The cellular functions of mechanosensory cilia within vascular system have emerged recently. In particular, hypertension is insidious and remains a continuous problem that evolves during the course of polycystic kidney disease (PKD). The basic and clinical perspectives on primary cilia are discussed with regard to the pathogenesis of hypertension in PKD.

## 1. Introduction

The changes in blood vessel diameter serve as an important physiological regulator of blood flow. These changes, caused by contraction and relaxation of vascular smooth muscle, can be regulated centrally and locally. The central regulation of cardiovascular function is achieved through neuronal control through complex projections from central and peripheral neurons [1–3]. The density of this neuronal innervation on the adventitial layer of blood vessels varies from tissue to tissue and among different vascular structures [4–6]. Thus, local regulation of the blood vessel becomes important, especially in vessels with less abundant innervation or central regulation.

The mechanism involved in local regulation of blood vessels is termed autoregulation. It is required to achieve an immediate control of blood flow within a specific region in the tissue. Autoregulation is an effective and efficient way to control the amount of blood flow locally without altering the neighboring systems significantly [7]. In an isolated blood vessel, it has been shown that a sudden increase of transmural pressure reduces vessel diameter [8–10], while a faster flow (higher shear stress) increases vessel diameter [9, 11–13]. All in all, the endothelial cells lining the lumen of the vessel have the ability to sense pressure and blood flow, and they are capable of transducing changes in mechanical forces into changes of vascular smooth muscle tone [14, 15]. Thus, endothelial cells are able to decrease and increase arterial diameter by altering contraction and relaxation behaviors of smooth muscle cells in the artery.

Biophysically, mechanical forces in the blood vessel can be observed in the forms of stretch due to distention of surrounding muscle, cyclic strain due to the pulsatile nature of blood flow, compression due to differential pressure along the vascular system, pressure due to surface force of the systolic blood flow, and shear stress due to drag force generated by the blood flow (Table 1). These forms of mechanical forces may be physiologically impossible to differentiate *in vivo* because of the complex nature of the cardiovascular system [16]. Yet, these forces are known to be distinct from one another in cell culture or *ex vivo* studies [17–20]. Understanding the short-term and long-term effects of each individual force can therefore provide a better understanding of cardiovascular response, remodeling, adaptation, and disease. Of the mechanical forces mentioned above, we will discuss biophysical shear stress, which is probably one of the most studied biophysical forces.

## 2. Primary Cilia as Fluid-Shear Stress Sensors

Primary cilia are usually classified as non-motile organelles with microtubules arranged in “9 + 0” fashion. It has been suggested that, like the nucleus, mitochondria, golgi, and other intracellular organelles, a primary cilium can also be viewed as a separate entity within a cell (Table 2) [21]. A cilium can be studied as an organelle with five distinct domains: (1) *the ciliary membrane*, a specialized domain composed of a protein and with a lipid composition different from that of the rest of the plasma membrane; (2) *the soluble compartment*, also known as the matrix compartment or cilioplasm; (3) *the axoneme*, composed of nine pairs of microtubules with a highly structured transport motor cytoskeleton; (4) *ciliary tip*, housed specialized proteins whose roles are still to be explored further; (5) *basal body*, a “mature” or “mother” centriole from which the primary cilium is projected.

**Figure 1:**
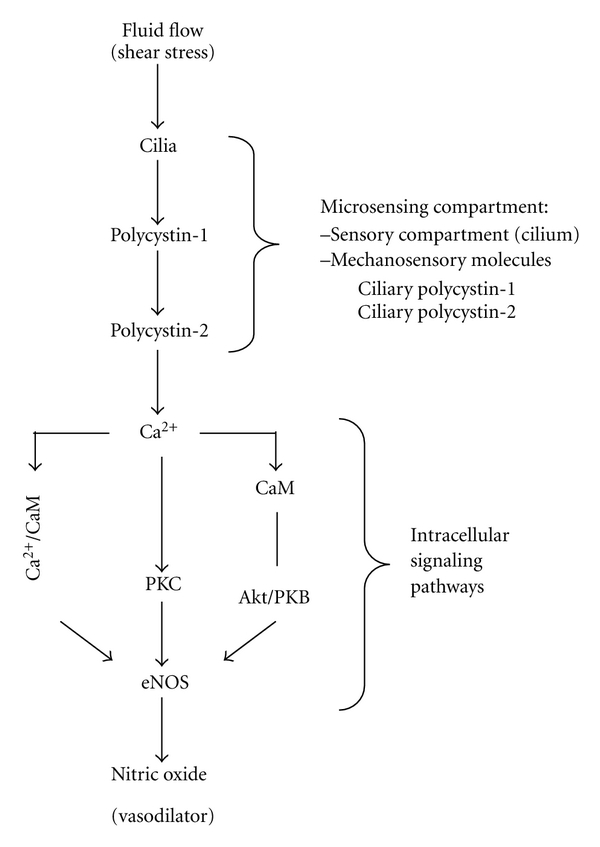
Mechanosensory cilia as microcompartments. Primary cilia are mechanosensory compartments that house many sensory proteins. Activation of these compartments through the sensory machineries will generate a cascade of various proteins activation, which results in nitric oxide production. CaM, calmodulin; PKC, calcium-dependent protein kinase; eNOS, endothelial nitric oxide synthase. Figure was adapted from [22].

As micro-sensory compartments, cilia have functions that depend on mechano-proteins such as polycystin-1 and polycystin-2 (Figure 1). Thus, the overall functions of the sensory compartments depend on both functional and structural cilia proteins. Within a blood vessel, an abrupt increase in blood pressure or shear stress can be detected by these sensory proteins localized in the cilia [16, 24]. With cilium functioning as a local regulatory mechanism, the extracellular fluid mechanics can then be transduced and translated into a complex of intracellular signaling, which in turn would activate eNOS—an endothelial enzyme that synthesizes nitric oxide (NO) gas. In particular, shear stress-induced calcium and NO signaling has been reported in many endothelial cells [25]. The released NO will diffuse from endothelial cells to the neighboring smooth muscle cells, thereby promoting vasodilation [26–28]. The overall effects of cilia function are thus to decrease total peripheral resistance, therefore lowering the blood pressure. 

The presence of primary cilia in vascular endothelia has been reported in human arteries [29–31] and has been observed in cultured human cells [16, 32, 33] and adult vascular system *in vivo* [34–38]. Of particular interest is a high level of polycystin expression in endothelial cells, which is required for the structural integrity of blood vessels [39–44]. The expression of polycystins in human endothelial cilia provides a critical link between cilia and the vasculature [16, 32, 33]. Interestingly, the function of polycystin-1 as a mechanosensory molecule can be inactivated by proteolytic cleavage after exposure to high fluid-shear stress. This indicates that cilia function can also be regulated through modification of polycystin-1 via a high shear stress [24]. This further suggests that in patients with high blood pressure, that is, high shear stress, cilia would very likely be unable to sense minute changes in blood pressure, which might result in failure to autoregulate the local circulatory system. This might increase the possibility of localized blood vessel injuries, such as aneurysm, atherosclerosis, dissection, edema, hemorrhage, and vascular ectasia, among others.

Throughout the cardiovascular system, patterns of fluid dynamics change considerably due to continuous vascular remodeling and patterning for microadaptation purposes [43, 45, 46]. The changes in the fluid dynamics generate differential biomechanical forces. These forces can initiate a complex of gene expressions [5, 7] which may also alter cilia function or structure in endothelial cells [24]. Consistent with this idea, it has been shown that not all vasculatures have cilia [38, 47, 48]. Only arteries with low fluid shear or high fluid turbulence have cilia, particularly longer, well-developed cilia. Because prolonged exposure to high fluid-shear stress would induce cilia to disassemble [33], it is possible that cilia may not be needed to sense high shear stress. Rather, endothelial cells may have other mechanisms, such as glycocalyx, to sense much higher mechanical forces [49, 50].

## 3. Converting Mechanical Sensor to NO Production

To test the hypothesis that cilia are mechanosensitive organelles, endothelial cells without cilia were isolated and generated from *Tg737* mouse. To further confirm that polycystin-1 and -2 are sensory proteins in cilia, endothelial cells derived from mouse and human with polycystic kidney were used. Ciliary polycystin-1 and/or -2 are absent from the primary cilia in these cells. In *Tg737* endothelial cells, polycystins are concentrated in the base of the primary cilia. Functional assays were carried out by challenging these cells with various magnitudes of fluid-shear stress (0.5–50 dyne/cm^2^). While shear-induced cytosolic calcium increase is observed in normal endothelial cells, neither endothelial cells' isolated mutants nor diseased arteries exhibit this calcium response to shear stress [16, 24]. 

To validate cilia roles in fluid sensing, endothelial cells or arteries were subject to various mechanical stimulations (Table 1). The specificity of cilia function is confirmed when the shear-*in*sensitive cells or arteries could respond to other mechanical stimulation. Most important is that PKD cells and arteries fail to produce nitric oxide (NO) in response to fluid-shear stress.

**Figure 2:**
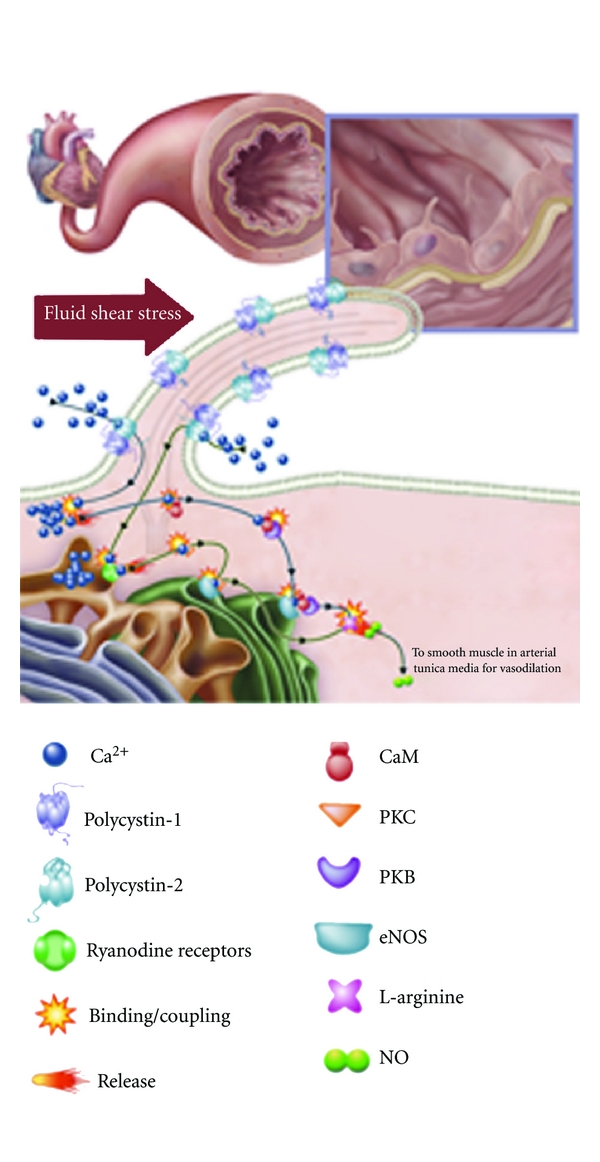
Mechanosensory cilia and nitric oxide production. The presence of cilia in vasculature plays an important role in the biochemical production of a potent vasodilator, nitric oxide (NO). The figure depicts the production of NO in an artery. Increases in blood pressure, which are translated to higher vascular shear stress, will be sensed by mechanosensory cilia. Bending or activation of the cilia involves mechanosensory polycystin-1 and polycystin-2 complex and a cascade of biochemical synthesis of NO. The cascade will further involve extracellular calcium influx (Ca^2+^), followed by activation of various calcium-dependent proteins, including calmodulin (CaM) and protein kinase C (PKC). Akt/PKB, CaM, and PKC are important downstream molecular components to activate endothelial nitric oxide synthase (eNOS). This figure is reproduced with permission [23].

To understand how ciliary polycystins are required to activate a biochemical cascade for NO production, various inhibitors were utilized to block the molecular functions [16]. Removing extracellular calcium with EGTA abolished both calcium and NO production in normal endothelial cells. Furthermore, L-NAME, an eNOS inhibitor, could block shear-induced NO biosynthesis, but not cytosolic calcium increase. This indicates that extracellular calcium influx is an upstream and prerequisite event. To explore calcium-dependent mechanisms of NO production, the roles of protein kinase C (PKC) and calmodulin were investigated with calphostin C and W7, respectively. Possible downstream effectors, including Akt, PKB, and PI3K, were examined with pharmacological blockers Akt inhibitor II, LY-294,002, and wortmannin. Interestingly, only Akt/PKB, but not PI3K, is involved in shear-induced NO production (Figure 2).

## 4. Pathogenesis of Hypertension in PKD

Polycystic kidney disease (PKD) is characterized by bilateral enlarged cystic kidneys, which have been associated with primary cilia dysfunction [22, 51, 52]. PKD is also characterized by various cardiovascular abnormalities. These abnormalities may include hypertension, cerebral and coronary artery aneurysms, mitral valve prolapse, aortic root dilation, dissection of the thoracic aorta, aneurysm formation in the abdominal aorta, vascular ectasia, and abnormal function of the microvascular bed [53–56]. Furthermore, the frequencies of cardiovascular complication in PKD patients are very high [57]. These include hypertension (78%), cardiac valve disorders (25%), and intracranial aneurysms (10%).

Hypertension, in particular, has been a continual risk factor for other cardiovascular complications in PKD. Similar to cystogenesis, pathogenesis of hypertension in PKD has also been associated with primary cilia dysfunction. Within the context of clinical hypertension, there are two theories that could help describe the pathogenesis of hypertension in PKD (Table 3). The first theory points to inherent cardiovascular dysfunctions as the primary cause of hypertension; the second theory brings about the cystic kidney itself as the origin for hypertension.

### 4.1. Cardiovascular Dysfunction as a Primary Factor

In an observational study of 312 children with PKD, it is reported that high blood pressure promotes faster renal volume growth. PKD children with high blood pressure have faster renal growth than those with lower blood pressure. This suggests that hypertension is a risk factor independent from kidney function in PKD [58]. Consistent with this view, it has been suggested that high blood pressure can actually promote faster cyst growth [59, 60].

Of note is that hypertension occurs at a much earlier age in patients with PKD than in the general population [61]. The median age for hypertension in PKD patients is about 30 years, compared with a median age of 45–55 years in patients with essential hypertension [62]. Hypertension occurs in children even before they are diagnosed with PKD [63–66] or before any substantial reduction in glomerular filtration rate is observed [67, 68].

To examine endothelial function in PKD, the plasma concentrations of vasodilator nitric oxide were measured in PKD patients and healthy controls. In this study, the plasma concentration of nitric oxide was reduced in PKD patients, confirming an association between PKD and endothelial dysfunction [69]. The endothelial dysfunction in PKD may thus be associated with abnormal cilia role in sensing fluid-shear stress and other downstream signaling mechanisms (Figure 2). To further substantiate the endothelial dysfunction in PKD, levels of ADMA (a marker of an inhibitor of nitric oxide synthase) were significantly increased in patients with early PKD compared to healthy age-matched individuals [70]. Although the significance of ADMA in PKD is not immediately understood, endothelia-dependent vasodilation offers substantial evidence which is too important to ignore.

Although ciliary therapy does not exist today, it is appealing and tempting to speculate the possibility of treatment for localized blood vessel injuries such as aneurysm, atherosclerosis, dissection, edema, hemorrhage, and vascular ectasia, among others in PKD. In particular, endothelium-dependent relaxation is impaired, and endothelial nitric oxide synthase activity is decreased in patients with PKD [71–73]. The endothelial dysfunction due to impaired release of NO in PKD patients becomes a crucial pathogenesis of hypertension. The imbalance in endothelium-derived vasoactive mediators might therefore need to be considered seriously in PKD patients [74, 75].

### 4.2. Cystic Kidney as a Primary Factor

**Figure 3:**
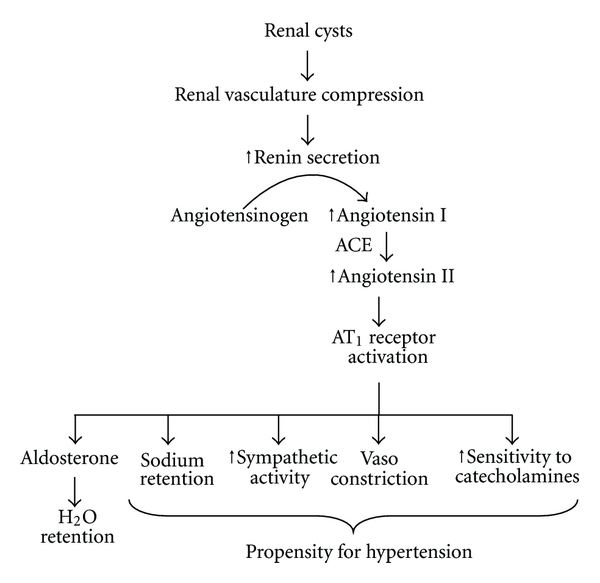
RAAS regulation in polycystic kidney disease. Renal cysts are thought to compress and disrupt the vascular network in the kidney. The kidney would then become ischemic, which would induce renin release from the juxtaglomerular apparatus. The increase in renin secretion could accelerate the conversion of angiotensinogen to angiotensin I, which is converted by angiotensin-converting enzyme (ACE) to angiotensin II. Activation of angiotensin receptor (AT_1_) would initiate cascades of physiological responses that would lead to hypertension.

It is believed that as renal cysts progress, the cysts will cause structural damage in the nephrons, which leads to distortion of the renal architecture (Figure 3). Such a distortion would compress the renal vasculature and attenuate the renal vessels, causing intrarenal ischemia and activation of the renin-angiotensin-aldosterone system (RAAS). Thus, as cysts enlarge, the RAAS is activated [76–78]. Not surprisingly, several studies have shown that the ACE-I (angiotensin-converting enzyme inhibitor) or the ARB (angiotensin receptor blocker) is effective in lowering blood pressure in PKD [79, 80].

Activation of the RAAS, which is well documented in the clinical course of the disease [81] and in PKD mouse models [82, 83], has been proposed to contribute to hypertension seen in PKD patients. RAAS activation has also been found in normotensive and hypertensive PKD patients, regardless of their blood pressure and renal function [84]. It is believed that the high level of circulating angiotensin II in PKD patients also contributes to the development of vascular hypertrophy, which is further implicated in vascular remodeling [85]. Changes in the vasculatures during the course of the PKD progression have therefore been observed in both human [86–90] and animal [91–95] studies. 

It was also reported that the sympathetic nerve activity is increased in hypertensive patients with PKD, regardless of renal function [96, 97]. This suggests that sympathetic hyperactivity could contribute to the pathogenesis of hypertension in PKD. However, it is not immediately understood whether the sensitivity to sympathetic nerve activity is a secondary effect due to an increase in RAAS system. In a 3-year prospective randomized double-blind study, ACE-I ramipril and the beta-blocker metoprolol were both effective for use as a first-line therapy in hypertensive PKD patients [98]. However, it was suggested that aggressive blood pressure control with these agents is necessary in order for them to be beneficial for PKD patients [99]. Of apparent complexity is that angiotensin can stimulate the sympathetic nervous system and that sympathetic nerve activity can also stimulate RAAS [100, 101]. At least in murine models of PKD, bilateral renal denervation could reduce cystic kidney size, cyst volume and most importantly, systolic blood pressure [102]. It is therefore very likely that sympathetic nerve activity would activate RAAS system, which would increase blood pressure. 

Since cardiovascular abnormalities are thought to initiate from the cystic kidneys in PKD, there has been a great interest in studying the outcomes after renal transplantation in these individuals. Interestingly, renal transplant recipients with PKD still showed an increase in cardiovascular morbidity as seen in non-PKD transplant recipients [103]. In a different report of eleven transplant cases in hypertensive PKD, only six patients showed improved blood pressure after transplantation [104]. Improved blood pressure was defined as the ability to reduce antihypertensive drug treatment after renal transplantation. These results suggest that while renal transplantation seems to have some beneficial outcomes, it is not sufficient to eradicate the hypertension in PKD patients.

## 5. Concluding Remarks

Primary cilium dysfunction has been associated with PKD, and primary cilia have also been proposed to regulate blood pressure. We believe that because our knowledge on cilia biology is still relatively limited compared to other organelles within a cell, there are certainly many more questions than answers that we could provide at present. In order to better understand the relationship between cilia and pathogenesis of hypertension, we need to understand the physiological roles of cilia in more detail and in many other organ systems. For example, vascular endothelial cilia have recently been proposed to regulate cell division [105] and endothelial-to-mesenchymal transition [106]. In addition, the presence of cilia in vascular smooth muscle cells has also been reported, and sensory polycystin-1 and polycystin-2 complex is localized in these cilia [107, 108]. Although their roles are not clear at present, the vascular cilia are positioned in such a way that they maintain a specific alignment with respect to the lumen of the artery. Further studies of the role of this alignment may be necessary to shed light on their possible functions with regard to vascular hypertension.

Whether or not dysfunction in primary cilia causes hypertension in PKD, there is certainly much work remaining. We are on the verge of applying our concept and understanding of PKD to better clinical practice and patient outcomes. Nonetheless, early and effective treatments of hypertension are clinically very important to decrease the morbidity and mortality of patients with PKD.

## Figures and Tables

**Table 1:** Mechanical forces in the blood vessel.

Types of force	Definitions
Stretch	Distention force by surrounding muscle
Cyclic strain	Pulsatile force by turbulent flow of blood
Compression	Contractile force by differential pressure in the vessel
Pressure	Systolic force on intima surface by kinetic flow of blood
Shear stress	Drag force along intima surface by kinetic flow of blood

**Table 2:** Five distinct domains of a cilium.

Domains	Functions
Ciliary membrane	Localization of chemo- and mechanosensory proteins
Soluble compartment	Localization of signaling molecules
Axoneme	Structural protein to support ciliary transport
Ciliary tip	Localization of specialized signaling molecules
Basal body	Network foundation for structural ciliary protein

**Table 3:** Pathogenesis of hypertension in PKD.

Theories	Descriptions
Inherent cardiovascular dysfunction (Figure 2)	(i) Ciliopathy (ii) Endothelial dysfunction (iii) Nitric oxide synthase dysfunction (iv) Increased sympathetic nerve activity

Secondary to renal cystic formation (Figure 3)	(i) Compression of renal vasculature releases renin (ii) Renin converts angiotensinogen to angiotensin (iii) Activation of angiotensin receptor will induce:
	(1) Vasoconstriction
	(2) Sensitivity to catecholamines
	(3) Salt retention, and so forth.
